# Correlates of stress are interactive and not unidimensional: Evidence from U.S. college students early in the COVID-19 pandemic

**DOI:** 10.1371/journal.pone.0271060

**Published:** 2023-04-17

**Authors:** Mahdi Rezapour, Matthew H. E. M. Browning, Lincoln R. Larson, Alessandro Rigolon

**Affiliations:** 1 Independent Researcher, Colorado, United States of America; 2 Department of Parks, Recreation and Tourism Management, Clemson University, Clemson, SC, United States of America; 3 Department of Parks, Recreation and Tourism Management, North Carolina State University, Raleigh, NC, United States of America; 4 Department of City and Metropolitan Planning, The University of Utah, Salt Lake City, UT, United States of America; National Cheng Kung University College of Medicine, TAIWAN

## Abstract

Studies have investigated various aspects of how the COVID-19 pandemic has impacted college students’ well-being. However, the complex relationships between stress and its correlates have received limited attention. Thus, the main objective of this study is to evaluate multiplicative associations between stress and demographic, lifestyle, and other negative emotion factors during the pandemic. We used data from a survey with 2,534 students enrolled in seven U.S. universities and analyzed such data with generalized additive Tobit models and pairwise interaction terms. The results highlighted associations and interactions between myriad factors such as students’ social class, income, parental education, body mass index (BMI), amount of exercise, and knowing infected people in the student’s communities. For instance, we found that the associations between feeling irritable and sad due to the pandemic were interactive, resulting in higher associated stress for students with higher levels of parents’ education. Furthermore, associations between taking precautionary actions (i.e., avoiding travel and large gatherings) and stress varied with the intensity of negative feelings (i.e., sadness and irritability). Considering these interaction terms, the results highlighted a great inequality in pandemic-related stress within low income, lower social class, and higher BMI students. This study is among the earliest that employed a stratified approach with numerous interaction terms to better understand the multiplicative associations between different factors during the COVID-19 pandemic.

## Introduction

SARS-CoV-2, which leads to the COVID-19 disease, spread rapidly around the world, sending billions of people into lockdown. There were many challenges that students faced, including concerns that outbreaks may adversely impact their grades, unexpected moves back to their parents’ homes, and delays in graduation and related ceremonies [1]. Increased substance abuse such as drinking alcohol has also been observed among college students during the pandemic [2].

The challenges of the pandemic can also be documented also from its impacts on students’ psychological and mental health. The pandemic has become an important source of stress for students due to factors such as isolation and changes in communication between teachers and students [3]. Therefore, it might be expected that stress levels among college students were elevated during the pandemic. However, studies on the associated factors of stress resulting from the early phase COVID-19 pandemic among college students remain limited. Some evidence can be garnered from studies in China focused on the general public, which found mental health issues including anxiety, depression, difficulty sleeping, and stress are some of the consequences of the COVID-19 pandemic [4, 5]. Also, Chinese researchers found that people younger than 35, students, and females faced worse mental health effects from the pandemic than others [4].

However, the majority of past studies have not focused on the associated factors of stress, and many did not focus on college students. Stress in college students is of great concern, given that approximately 80% of students have reported moderate levels of stress, and approximately 12% have reported severe stress levels even before the COVID-19 pandemic [6]. The implications can be seen in Europe, where a study found that 15% of students have suicidal thoughts [7], and 3% have suicidal tendencies [8]. Since academics are an integral part of college student’s life, without a healthy attitude to reach academic goals, students’ entire college experience is expected to be impacted by their stress levels and mental health [9].

Numerous studies have been conducted on the correlates of stress in college students prior to the COVID-19 pandemic. Stress, sex differences, and coping strategies among college students have been found to have interactive effects [10]. Female college students tend to report higher overall stress levels and greater use of emotion-focused coping strategies than male students. Another study of the prevalence and correlates of depression, anxiety, and stress among college students found levels were higher among off-campus and transfer upperclassmen students [11]. College student stress levels have also been related to binge-watching, online shopping, and sexing [12]. Anxiety mediated the pathways between some source of strain and binge-watching and online shopping [12].

Limited studies have also been conducted on associations between stress in college students during the early phase COVID-19 pandemic. In particular, the impact of the pandemic on higher education was evaluated from the psychosocial context of positive and negative impacts [13]. The results highlighted that students were among those that suffered the most as a result of the pandemic, in terms of factors such as temporary employment regulation and negative impacts from teleworking, with some of these impacts more severe in women than men. Ultimately, despite the importance of studying the complex interactions within correlates of students’ emotional and mental health, the majority of past research has only considered how stress was associated with or caused by demographic and lifestyle factors under the assumption that these factors were stable across levels of other variables of interest.

Studies also conducted highlighting the significance of studying individuals not only across different context of careers, e.g., students, but also context of culture and nationalities, where few studies will be highlighted here. Variations across international and local students, in response to the COVID-19, in terms of anxiety and suicidal ideation were evaluated. The results highlighted that local student have decreased sufficiency with resources and anxiety compared with international students. in another study staying with family members, satisfaction with support and information seeking were some of the important variables at the time of COVID-19 in predicting the level of anxiety across students [14]. Also, the importance of giving especial care to students with particular cultural background due to the differences at the time of pandemic was observed in the past study [15].

The current study challenges the assumption that associations between various attributes and stress are stable; rather, it investigates whether these associations might vary by students’ demographics, negative emotions, and behavioral factors. This study, therefore, sets forth to focus on the main question of whether associations between various negative emotions such as feeling sad and irritable remain stable across various levels of stress or whether these associations are multiplicative. The confounding effects and pairwise interaction terms across all demographic and lifestyle variables are considered. We employed interaction terms to answer our main research questions: first, do associations between various negative emotions and stress due to the pandemic vary across students with various socioeconomic characteristics; and second, are associations between demographic and lifestyle predictors and stress interactive? Such interactions between socioeconomic characteristics of students and taking precautionary actions are important due to the potential for buffering effects of those factors.

The unprecedented events of the COVID-19 pandemic have not ended. Further, they might re-occur in the future due to future pandemics or the worsening of other global challenges (i.e., the climate crisis). Based on the above discussion, it is expected that college students to be one of the most vulnerable groups to be impacted by these events. Therefore, the current pandemic provides an unprecedented opportunity to investigate the behavioral and emotional health of students. The results of the current study hope to prepare universities and mental health professionals to tailor appropriate actions to the specific need of students based on their characteristics.

## Method

### Data analysis

We used generalized additive Tobit models to analyze the data. Such models are useful when there is a concentration of observational points at limit boundaries. That was applicable for our study since response options were bounded (i.e., 0 to 100). There is the possibility of having negative or positive deviations from those values, however [16]. Correspondingly, we used Tobit models as we expected many respondents had some positive or negative deviations from the extreme points. In other words, many respondents selected the first or last alternatives, while they believed in more extreme options. As a result, the upper limit (100) and lower limit (0) were expected to be dissimilar across respondents. Here, the response is feeling stress due to COVID-19 on college students. With $P(Y=L)=P(Y^{*}\leq L)$ and $P(Y=U)=P(Y^{*}\geq U)$, the likelihood of the Tobit model is:

Equation 1:


$$\prod_{y_{i}=L}\Phi (\frac{L-\mu_{i}^{*}}{\sigma_{i}})\prod_{L<y_{i}<U}\frac{1}{\sigma_{i}}\phi (\frac{y_{i}-\mu_{i}^{*}}{\sigma_{i}})\prod_{y_{i}=U}\Phi (\frac{-(U-\mu_{i}^{*})}{\sigma_{i}})$$ 1

Where Φ and *ϕ*, are Gaussian cumulative distribution function (CDF) and probability density function (PDF), respectively. As can be seen from Eq 1, the likelihood function comprises a mixture of two discrete parts and one continuous part in the middle. Also, given $\mu_{i}^{*}=x_{i}^{⊺}\beta +\varepsilon_{i},\;where\;\varepsilon_{i}∼N(0,\sigma^{2}),$ it is clear that while getting the log likelihood (*LL*) of Eq 1, the final *LL* is the sum of three expressions across all observations: those at the limits of *U* and *L*, and those for <*y*_*i*_<*U*. There are a few types of the Tobit model. Here we employed uncensored Tobit models since no values were censored from the dataset.

Generalized additive models (GAM) were used to account for possible non-linear relationships between the response and predictors. Smoothers in GAMs allow a data-driven approach to the model-driven model process [17]. The objective function of the model could be written as a minimizer of the objective function as:

Equation 2:


$$S(f)=\sum_{i=1}^{n}{\left(y_{i}-f(x_{i})\right)}^{2}+\lambda \int_{a}^{b}{\left\{f^{''}(x)\right\}}^{2}dx,$$ 2

Where *f*(*x*_*i*_) is a smooth function, *λ* is a smoothing parameter, which could be used to control a model’s smoothness, and *f*′′(*x*) is the second derivative of the smooth function to account/penalize for the wiggliness of *f*(*x*_*i*_).

As can be seen from Eq 2, while the first expression on the right hand side (RHS) uses all values of *i*, the second expression uses only the smooths: *a*<*x*_1_<⋯<*x*_2_<*b*. It is also clear that the first expression in the RHS of Eq 2 is related to the residual sum of squares, while the second term penalizes for lack of smoothness. To remedy this, we used the backfitting technique [18], which iteratively estimates a single smoother component, one at a time, though partial residuals.

B-spline basis functions were used as the basis for the vector space. Cubic splines were written as linear combinations of these basis functions. The objective function of the cubic smoothing spline, based on the penalized least square Eq 2, was written as:

Equation 3:


$$s(f)={\left(y-f\right)}^{⊺}\Sigma^{-1}(y-f)+\lambda f^{⊺}kf$$ 3

Where *k* is a roughness penalty matrix, and after setting its derivative to 0, we have:

$$\hat{f}=S(\lambda )y$$ 4

And where the influence matrix of *S*(*λ*) was written as:

$$S(\lambda )={\left(I_{n}+\lambda \Sigma K\right)}^{-1}$$ 5

Here, *Σ* was written based on the weights, *W*, as *Σ* = *W*^−1^. We smoothed two parameters of worry about COVID-19 and limiting exercise at the gym by cubic splines (see Table 2). No more predictors were considered to be smoothed due to a lack of interpretability. In this study, we considered and checked all interaction terms between included variables. We limited ourselves to pairwise interactions terms due to the complexity in interpreting 3-term interaction terms by general audiences. The improvement in the model fit by means of VGAM and interaction terms was evaluated by means of Akaike’s information criteria (AIC), which penalized for large numbers of incorporated parameters. For a discussion on the importance of considering interaction terms in general, please refer to past research [19].

### Data

Data were collected from a study of 2,534 college students at seven large public universities across the U.S. in the early stages of the COVID-19 pandemic [20]. Human subject approval was awarded at each institution including Clemson, North Carolina, and Utah university, where the method was carried out in accordance with relevant guidelines and regulations. It should be noted that the data were collected in spring 2020, when the first and most severe real lockdown occurred in the U.S. Several points are worth mentioning here regarding the sources of the survey items, which the next few paragraphs will outline.

#### PANAS

Negative emotions, such as being afraid, irritable, guilty, and sad, were based on the development of the positive and negative affect schedule (PANAS) [21]. Feeling stress was part of the negative emotions that were considered. In the source article, participant burden was minimized by using items answered on the visual analog scale (VAS) [22]. These questions related to how much time students thought about the pandemic, where a concept derived from the eating disorder literature [23].

Some of survey items measured the concepts such as negative emotion states, preoccupation with COVID-19, feeling stressed, worry, and time demands. For coming up with the items, the original study examined studies of other large-scale disasters (i.e., the World Trade Center terrorist attacks on September 11, 2001), which are associated with psychological impacts on the general population [24].

#### Sociodemographic factors

Based on the past research, numerous risk factors such as race, gender, age, perceived social class, academic status, parental education, and relative family income were included in the survey as well. Socioeconomic status (SES) variables included seven questions on class, parental education, and relative family income [25]. To measure academic status, the respondents were asked whether they were in pursuit of an undergraduate or graduate degree.

#### Lifestyle-related

General health from poor to excellent [26] and BMI were measured as potential confounding factors for COVID-19 [27]. BMI was measured from self-reported students’ height and weight. Time spent on screens or performing exercise were included as possible lifestyle-related factors [28]. Here, the lifestyle factors related to time outdoors included the number of hours in the last day spent in parks, on greenway trails, etc. "Screens" included using a smartphone, computer, watching television, online gaming, etc.

Likert-type response scales with strongly agree-strongly disagree anchors ranged from 1 and 5 or 1 and 7. The lowest values for ordinal and binary variables were considered as the reference categories. The response variable was the amount of stress due to the COVID-19 pandemic that the students experienced. Negative emotions based on the PANAS were measured on a 1–100 response scale.

#### Others

Time demands were measured by the original study [19]. Those questions were developed from survey prompts in the eating disorder literature [23]. Specifically, the original study asked to what extent respondents believed they spent time on the pandemic. For that, a 5-point Likert-type agree-disagree response scale was used. As a potential risk factor, we asked from the respondents if they know people who were diagnosed with the virus, e.g., someone in their family or someone in their community [29].

## Results

Two models were compared in terms of goodness of fit, including the generalized additive Tobit and the standard Tobit model. Here, after presenting the data summary, we briefly compare the considered models in terms of goodness of fit (Table 2). In addition, Table 2 incorporates two models, with and without the interaction terms. Then we present the results in terms of their main and interaction terms.

### Descriptive summary

The survey asked students a variety of questions about their feelings and behaviors related to the pandemic. A descriptive summary of the key variables is presented in Table 1, and detailed descriptions of the measures can be found in the source article [20]. All subjects provided informed consent while completing the online survey.

**Table 1:** Variables incorporated in statistical analysis.

	Mean	Variance	Min	Max
How stressed do you feel when you think about COVID-19?	64.03	693.916	0	100
Precautionary actions: Limiting exercise at the gym	4.65	0.938	1	5
Precautionary actions: Avoiding large groups of people	4.16	0.794	1	5
Precautionary actions: Avoiding travel	4.52	0.616	1	5
General health	3.32	1.031	1	5
How worried do you feel when you think about coronavirus?	4.03	2.769	1	7
Spending too much time thinking about COVID-19	3.98	2.959	1	7
How afraid do you feel when you think about COVID-19?	50.55	741.890	0	100
How irritable do you feel when you think about COVID-19?	59.43	791.258	0	100
How guilty do you feel when you think about COVID-19?	24.36	657.680	0	100
How sad do you feel when you think about COVID-19?	61.12	725.576	0	100
Number of hours spent in front of a screen during the last 24 hours	7.74	7.207	0	12
Hours have been spent outdoors during the last 24 hours	1.58	2.580	0	12
Number of hours spent doing exercise during the last 24 hours	0.95	1.158	0	12
Highest level of dad education	4.54	2.656	1	7
Income relative to others	3.31	1.224	1	5
Social class	2.82	1.037	1	5
Knowing infected person(s) in the local community	0.25	0.176	0	1
Gender: male* vs. female	0.61	0.233	0	1
BMI	24.11	21.066	5.4	60.1
Age: 18–24 years old vs. others*	0.23	0.178	0	1
Highest level of mother education	4.51	2.250	1	7

*Reference category is the lower value

### Model goodness of fit

The generalized additive Tobit model, AIC = 19,721 (Log-Lik = -9,822), outperformed the standard Tobit model, AIC = 17,742 (Log-Lik = -9,839). Considering models with and without interaction terms, major differences could be observed in terms of statistical significance levels and magnitudes of the variables’ coefficients, especially related to the interaction terms (Table 2). For instance, while originally considering only the main effects of variables, measures of parental education were not different from zero, but they were significant for the model considering the interaction term. This finding highlights the impact of interaction terms in the model’s goodness of fit and value in considering interaction terms in expanding our understanding of the real impacts on the response variable.

**Table 2:** Parameters estimates of models with and without interaction terms.

	*With interaction terms* ^*a*^			*Without interaction terms* ^*b*^		
*Parameters*	*Coef*.	*SE*	*p-value*	*Coef*.	*SE*	*p-value*
(Intercept):1	16.01	9.133	0.080	12.74	4.263	<0.005
(Intercept):2	2.85	0.015	<0.005	2.86	0.015	<0.005
***Students’ characteristics***						
Gender, Female	2.26	0.762	0.003	2.34	0.767	<0.005
General health	-2.80	0.381	0.000	-2.83	0.383	<0.005
Social class	-4.91	1.635	0.003	-1.81	0.445	<0.005
Age	-6.68	2.521	0.008	-1.23	0.902	0.2
BMI	0.47	0.197	0.018	0.12	0.082	0.16
Income relative to others	-1.31	1.016	0.198	0.56	0.424	0.19
***Parental characteristics***						
Highest level of mother education	-0.99	0.563	0.080	0.03	0.285	0.9
Highest level of dad education	0.94	0.574	0.102	-0.24	0.268	0.4
***Behaviors during COVID-19***						
Spending too much time thinking about COVID-19	3.30	1.118	0.003	1.70	0.274	<0.005
Number of hours spent doing exercise	-2.29	0.786	<0.005	-0.85	0.398	0.03
Number of hours spent in front of a screen	-0.77	0.425	0.071	0.17	0.144	0.2
Precautionary actions: avoiding travel	2.80	0.924	<0.005	1.07	0.524	0.04
Precautionary actions: avoiding large groups of people	-3.34	1.154	<0.005	-1.17	0.469	0.01
***Feelings related to COVID-19***						
Feeling guilty due to COVID-19	0.04	0.015	0.016	0.04	0.016	0.01
Feeling afraid due to COVID-19	0.31	0.019	<0.005	0.31	0.019	<0.005
Feeling sad due to COVID-19	0.47	0.076	<0.005	0.24	0.018	<0.005
Feeling irritable due to COVID-19	0.09	0.041	0.023	0.20	0.014	<0.005
***Other factors***						
Infected person in the local community	-5.41	2.083	0.009	0.33	0.858	0.7
Hours have been spent outdoors	0.48	0.275	0.084	0.58	0.276	0.03
***Interaction terms including negative feelings***						
Feeling irritable due to COVID-19× Highest level of mother education	0.02	0.008	0.019	-----	-----	-----
Feeling sad due to COVID-19× Highest level of dad education	-0.02	0.008	0.017	-----	-----	-----
Feeling sad due to COVID-19× Precautionary actions: avoiding travel	-0.03	0.015	0.028	-----	-----	-----
Feeling irritable due to COVID-19× Infected person in the local community	0.09	0.031	0.002	-----	-----	-----
***Other interaction terms***						
Spending too much time thinking about COVID-19 × BMI	-0.08	0.044	0.053	-----	-----	-----
Spending too much time thinking about COVID-19 × Number of hours spent doing exercise	0.44	0.204	0.031	-----	-----	-----
Social class × Precautionary actions: avoiding large groups of people	0.75	0.379	0.049	-----	-----	-----
Number of hours spent in front of screen × Income relative to others	0.23	0.118	0.047	-----	-----	-----
Number of hours spent in front of screen × Age	0.71	0.300	0.018	-----	-----	-----
*Smoothers*	*Coef*.	*DF*	*p-value*			
S (I worry about COVID-19all the time)	2.64	3	<0.005	2.66	0.325	<0.005
S (Limiting exercise at the gym)	-0.12	3	0.053	0.07	0.385	0.9

Model considering all important interaction terms.

Model with no interaction term to hight the difference with model ‘a’.

### Main effects

While we considered interaction terms, we observed some main effects worthy of discussion. These included students’ gender, general health, and feelings related to COVID-19, including feeling guilty and afraid, as well as lifestyle factors (i.e., time outdoors).

### Students’ characteristics and feelings related to COVID-19

The main effects of gender and the general health of students were found to be important. Female students, ${\hat{\beta }}_{Gender}=2.26$, felt more stress due to COVID-19 than their male counterparts. Students who reported better general health, ${\hat{\beta }}_{General\;health}=-2.80$ felt less stress due to COVID-19 than students in poorer health. This association might be explained by the characteristics of healthy students coping and taking a "growth mindset" approach to stressors. Negative feelings of guilt, ${\hat{\beta }}_{guilty}=0.04$, and being afraid due to COVID-19, ${\hat{\beta }}_{Afraid}=0.31$ were both positively associated with stress due to COVID-19.

### Interaction terms

As we incorporated the interaction terms, we no longer could solely interpret the main effects unless the impacts of the main effects were so large that they covered the impact of the interaction terms. To organize the interaction terms, they were divided into two main groups: those that included negative feelings and others.

#### Interaction terms including negative feelings

The education levels of students’ mothers and fathers were found to be important in shaping the level of stress that students experienced due to COVID-19. The estimated coeffects (${\hat{\beta }}_{Mother\;education}=-0.99,\;{\hat{\beta }}_{Irratible}=0.09$) highlighted that the associations between feeling irritable due to COVID-19 and feeling stress were multiplicative, indicating that students’ feelings of irritability were much lower across those with lower maternal education levels compared with among those with higher maternal education levels. It could be seen that the effect of maternal education, ${\hat{\beta }}_{Mom\;education}=-0.99$, was so larger that it covered the impacts of feeling irritable and the related interaction term, ${\hat{\beta }}_{irratable\times Mom\;education}=0.02$. Although similar to maternal educations, the association between paternal education and stress was multiplicative; students with higher paternal education levels experienced more stress: ${\hat{\beta }}_{sad}=0.47$, ${\hat{\beta }}_{sad\times dad\;education}=-0.02$, and ${\hat{\beta }}_{Dad\;education}=0.94$.

We observed that associations between feeling sad and stress were dependent on the degree of limiting travel, ${\hat{\beta }}_{sad}=0.47,\;{\hat{\beta }}_{travel\;plan}=2.80,\;{\hat{\beta }}_{sad\times travel\;plan}=-0.03$. The results highlighted that there was a larger positive association between feeling sad and stress across students that limited travel.

The impact of knowing someone infected with COVID-19, ${\hat{\beta }}_{infected\;any}$ = -5.41, was in tandem with positive coefficients of feeling irritable, ${\hat{\beta }}_{irritable}=0.09$, and interaction terms, ${\hat{\beta }}_{irritable\times infected\;any}=0.09$. However, as can be seen from the estimated coefficients, the magnitude of knowing infected persons was much larger.

#### Other interaction terms

An interaction between body mass index (BMI) and thinking too much about COVID-19, ${\hat{\beta }}_{too\;much\;time}=3.30,\;{\hat{\beta }}_{BMI}=0.47,\;{\hat{\beta }}_{too\;much\;time\times BMI}=-0.08$, highlights that the positive association between thinking too much about COVID-19 and stress was stronger among students with higher BMI. Although the interaction term was in tandem with the main effects, the values of the main effects were much higher.

Another significant interaction term was related to the sense of spending too much time thinking about COVID-19 and hours of exercise (${\hat{\beta }}_{too-much\;time}=3.30,\;{\hat{\beta }}_{hours\;of\;exercise}=-2.29,\;{\hat{\beta }}_{too-much\;time\times hours\;of\;exercise}=0.44$). In general, while the two variables interacted with each other to influence stress, they were in tandem. Magnitudes of the point estimates highlighted that students who felt they spent too much time thinking about COVID-19 had higher levels of stress.

The association between stress and avoiding large groups (${\hat{\beta }}_{avoiding\;large\;groups}=-3.34$), varied by students’ perceived social class, ${\hat{\beta }}_{social\;class}=-4.91$. Students who felt they belonged to a higher social class and avoided gatherings felt less stress. Based on these interaction results, it is worth highlighting that students reporting a higher social class might take stricter measures in limiting gatherings and therefore undergo lower levels of stress than other students.

There was also an interaction term between the perceived relative income of students and amount of time on screens, ${\hat{\beta }}_{screen}=-0.77,\;{\hat{\beta }}_{income}=-1.31,\;{\hat{\beta }}_{screen\times income}=0.23$. More screen time and higher relative income were both associated with less stress. Although similarities could be noted between social status and income, social status is more comprehensive as it is a combination of factors such as income and occupation [30].

The next interaction term worthy of discussion is related to screen time and age (${\hat{\beta }}_{Age}=-6.68,\;{\hat{\beta }}_{screen\times Age}=0.71$). As might be expected, the association between screen time and stress varied based on the age of students. In general, older ages and more screen time were associated with less stress. It should be highlighted that the age measured seemed to be much more significant in the reduction of stress than screen time.

### Summary of model results

**Fig 1:**
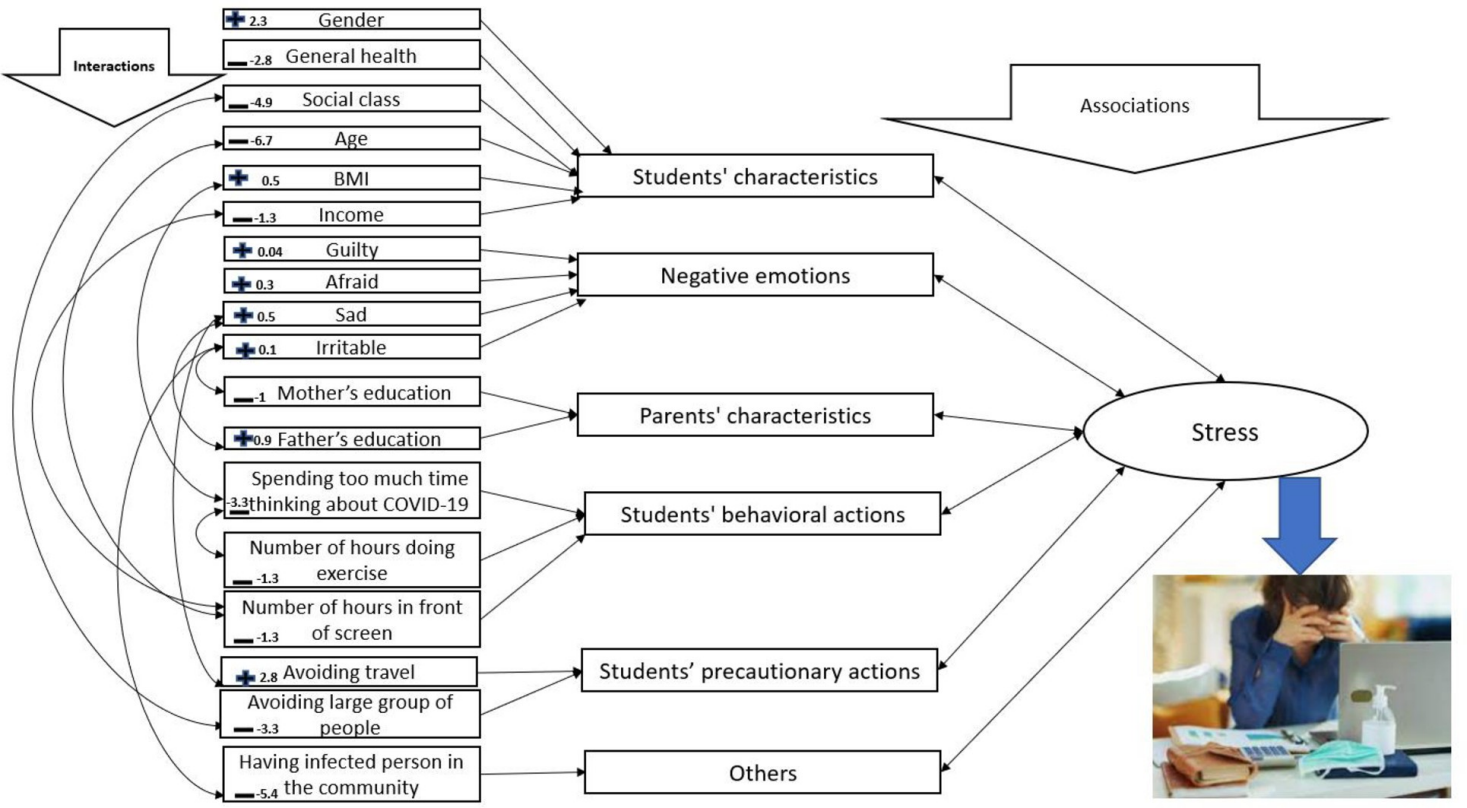
Summary of the complex relationships between college students’ stress from the COVID-19 pandemic (left hand side) and associations with myriad factors (right hand side); positive and negative signs show whether associations with more stress are positive or negative.

The results in Table 2 are depicted in Fig 1 to provide a better perception of the associations between various factors and feeling stressed as the response. The arrows on the left hand side correspond to interaction terms, while double arrows on the right hand side relate to the associations. Although interactions do not mean a sole impact of particular variables on the response, they highlight associations between stress and demographic, lifestyle, and other negative emotions factors with positive (+) and negative signs (-) to highlight whether higher or lower values of those factors are associated with more stress. For instance, the higher category for gender (female students) showed a positive sign, so it was associated with higher stress. Another example is for social class; higher social classes showed a negative sign and were associated with less stress. It should be noted that although the magnitudes of coefficients were included, they should be interpreted in their own context. For instance, gender is binary, while various negative emotions are on the scale of 0–100. That is why the magnitudes of various negative emotions are much smaller than gender.

## Discussion

Although correlates of stress have been previously examined, most research has ignored the likelihood of multiplicative interactions between these variables and instead only considered their main effects. Considering the interaction terms is important as they expand our understanding of students being one of the most vulnerable groups impacted by the COVID-19 pandemic.

Our findings revealed that while intrapersonal factors such as higher social class, income, maternal education help buffer against COVID-19 related stress, factors such as various negative emotions, higher BMI, younger ages, and being female can offset those benefits. Also, there we observed associations between stress and various behavioral actions, such as limiting travel, engaging in exercise, avoiding large groups, and spending time on screens.

The associations between only a few variables and stress were found to be stable. For instance, female students and students with poorer general health experienced more stress than their counterparts. However, the associations between the majority of factors were not additive but varied based on other predictors. For instance, the association between thinking too much about COVID-19 and stress was not constant but varied by BMI. More importantly, the association between taking precautionary actions and stress varied by income. It was also interesting to see that, while feeling irritable was positively associated with stress, the irritability-stress gradient was steeper for students with lower maternal education levels. Those relations would be obscured if we had employed a conventional way of considering only the main effects.

These findings point to some potential takeaways that could be employed by policymakers and researchers interested in public health promotion amongst college students. While implementing policies, stratifications due to the presence of population structures are recommended. That is, significant systematic differences were present across several variables. For instance, the association between precautionary actions and stress varied by income and social class. These impacts are likely to be due to a lack of financial support among lower-income students and that many students and parents lost their jobs or sources of income during the pandemic [31].

Although variations across parental impacts with opposing directions of effects might seem counterintuitive, studies investigating the variations across the roles of parents on students might explain these findings. There is limited study of the impacts of parents on stress levels; here we make a contrast with studies that investigated impacts of parents on other aspects of students. For instance, the differential impacts of fathers’ and mothers’ involvement in students’ achievement have been investigated [32]. In that study, differences were observed across the two figure’s responsibilities. For instance, maternal school involvement was positively related to student achievement while the reverse was held for paternal involvement. In another study, the father’s influence on students was independent and over and above that of mothers [33]. Although students’ achievement is different than the stress measure considered in this study, the comparison may still represent differences in the impacts of these two figures on generally students’ lives at college.

The results highlight that knowing an infected person(s) correlated with less stress due to COVID-19. The results could be expected, since knowing infected individuals might provide realistic fear and stress about the serious conditions that might impact the student respondents as well.

Also, based on the point estimates, restricted travel was associated with stress to a greater magnitude than sadness itself. The importance of travel has been examined from different perspectives, from psychological wellness [34] and emotions [35] to recovery from stressors [36]. The relationship between sadness and stress has also been identified in numerous past studies [37]. However, none of those studies looked at the interactive relationship between stress and sadness or banning travel, which limits the ability to interpret these results.

On the other hand, engaging with higher levels of exercise was associated with students experiencing less stress. This result is somewhat in line with past research, which found that self-reported regular exercise resulted in fewer negative emotional consequences due to stress [38]. Also, interactions between negative feelings and senses of passing time were highlighted in a previous study which found anxious people subjectively experienced time moving more slowly [39]. Also, the significant relationship between time perception and anxiety in adolescents has been observed in past studies [40]. However, none of the above studies considered interactions between the amount of exercise and spending too much time thinking about an issue.

In general, this relationship between screen and stress somewhat conflicts with past research, which found stressed college students reported more screen time [41]. However, the context of this COVID-19 study should be taken into consideration. Age differences in coping with stressful incidents might be a result of variations in what people were expected to cope with when they age [42]. Coping can also be referred to the thoughts and behaviors of individuals managing or handling the burdens of an incident. For instance, at older ages, while individuals are likely to cope more with illness, or married lives, at younger ages they deal with other matters. Finally, the factors of worrying about COVID-19 and limiting the amount of exercise were found that should be smoothed instead of modeled linearly with the response.

Interactions between social class and taking precautionary actions could also be looked at from the angle that social class perceptions impact a variety of social tendencies, from causal attribution to moral judgment [43]. Also, our finding that men felt less stressed than women could be linked to gender differences in emotional responses to the pandemic [44].

We also found that the association between stress and thinking too much about COVID-19 varied by BMI and hours spent exercising. This finding highlights the importance of healthy weight status and moderate/vigorous physical activity to reduce stress during but not limited to the COVID-19 pandemic. This pandemic has resulted in the closure of many public places and fitness facilities. Universities and organizations should be better-prepared and dedicated to organizing exercise opportunities for students so they can remain active while taking into consideration safety measures, like social distancing.

Lastly, the association between stress and feeling irritable was found to vary by whether students knew someone infected with COVID-19 in their community. This finding is likely due to a lack of understanding regarding the consequences of being infected by the disease. Media plays a central role in communicating and educating students [45] so students’ perceptions about the disease could be better shaped, so stress levels are not elevated.

This study has some limitations worth noting. First, we did not consider 3-term interaction terms. There is a likelihood that many of these interactions were important. We did not consider those terms due to the higher complexity of terms and challenges for the general audience. Second, the observations were limited to college students. Although the questions were designed for that group, caution should be given to extrapolating our results to other audiences. Third, BMI was an important predictor that we used in the analysis. However, it has been found that both genders are expected to overestimate their height and underestimate their weight [46]. In our defense, alternative measures of underweight/overweight status using web-based surveys were not available or may have resulted in even less robust measures. In-person interviews or medical records to validate BMI with accurate weight statuses are recommended in future research.

Despite these limitations, we found suggestive evidence that associations between various emotions, behaviors, demographic characteristics, and stress should be considered interactively. Considering the interaction terms, our findings highlighted a great inequality in terms of associated stress across lower levels of income and social class as well as higher levels of BMI. The multiplicative effects highlight that even the associations between stress, as the response, and negative emotion such as feeling sad varied by precautionary actions.

The method outlined in this study could be employed in future research to better understand the complex associations between stress and its correlates. Such an approach is especially important in analyzing data unidimensionally that would obscure results. Due to the complex nature of stress across students, monitoring and follow-up with students are recommended to better understand their emotions and behaviors during and after the COVID-19 pandemic.

## Conclusion

The COVID-19 was used in thus study as a case study to see if the correlates of stress and various factors are interactive or unidimensional. All pairwise interaction terms across all predictors were considered. The results highlighted that the relationship between stress and almost all factors are complex and multidimensional.

On the other hand, considering a model with no interaction term highlighted that ignoring those interaction terms would result in biased or even erroneous point estimates, and thus future studies are encouraged to take into account the complex associations of various factors while modeling stress.
